# Integrated device for plasma separation and nucleic acid extraction from whole blood toward point-of-care detection of bloodborne pathogens†

**DOI:** 10.1039/d4lc00571f

**Published:** 2024-10-18

**Authors:** Abigail G. Ayers, Christia M. Victoriano, Samuel K. Sia

**Affiliations:** a Department of Biomedical Engineering, Columbia University New York NY 10027 USA ss2735@columbia.edu

## Abstract

Sample preparation presents a major challenge in point-of-care (POC) diagnostic assays, including ones requiring whole blood as the starting specimen. This study presents an integrated sample preparation device – which we call PRECISE – that performs both plasma separation and nucleic acid extraction, enabling streamlined sample preparation from whole blood requiring only a commercially available blood collection tool and a syringe, and no other external equipment or electricity. Plasma separation is performed using a dual-membrane filter (which filters out blood components while limiting membrane clogging) integrated into the cartridge, and nucleic acid extraction is performed by users moving magnets (to mix the samples, and along a guided track). The plasma filtration demonstrated recovery on par with lab-based centrifugation, and the extraction module showed performance similar to benchtop-based magnetic bead extraction. A sample-to-result demonstration on 50 μL of whole blood spiked with virions of hepatitis C virus (HCV), operating the PRECISE cartridge in 16 minutes followed by benchtop PCR, showed a limit of detection (∼6770 IU mL^−1^) on the order of the minimal requirements of target product profile for POC HCV detection. Future work on the PRECISE cartridge, building on POC accessibility and fast sample preparation demonstrated in this work, may enable detection of bloodborne pathogens from whole-blood specimens collected at the POC.

## Abstract 2

This work presents PRECISE, a device that integrates plasma separation and nucleic acid extraction, enabling streamlined sample preparation of whole blood for point-of-care diagnostics without external equipment or electricity.

## Graphical abstract

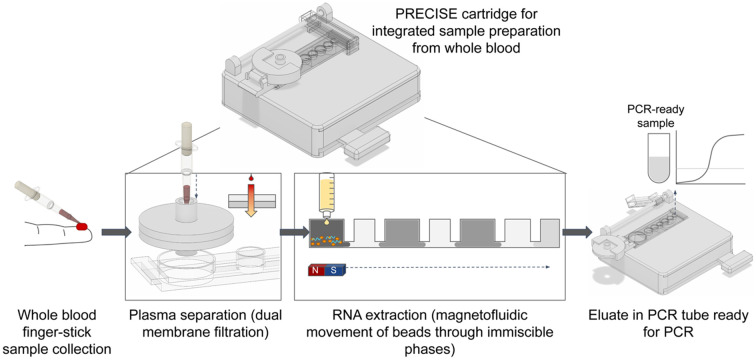

**Sam Sia's tribute to George Whitesides**

Growing up, I was entranced by the tales of James Watson and Richard Feynman. A dream came true when I had a chance to work with George Whitesides, a similarly brilliant mind. I wrote a proposal to the Canadian Institutes of Health Research to develop a microfluidic immunoassay, at a time (in 2002) when biological assays were just beginning to be constructed using the lab-on-a-chip toolbox. George told me, along with another postdoctoral fellow Vincent Linder, about his interest in improving global health, and his fascination with paper as a substrate for microfluidics. George called this line of work “Simple Solutions”. In a draft of a manuscript, I sheepishly tucked the global health focus into a corner of the manuscript; George advised me to put global health front and center if that was what was driving all of us to do the work. Other ideas percolated over coffees in the Science Center: how the petrochemical economy works (as sketched on a napkin), to the (then) underexplored mitochondrion. I am grateful to be in the orbit of George to receive a most precious gift: an experience of how the best, most creative, and inspiring science is meant to be. Samuel Sia

## Introduction

Sample preparation presents a major challenge in point-of-care (POC) diagnostic assays, including the detection of nucleic acids from bloodborne pathogens. In whole-blood specimens, blood cells can interfere with the accuracy of nucleic acid detection.^1^ As such, nucleic acid detection assays typically require plasma or serum, obtained *via* separation from whole blood using techniques such as centrifugation, which requires expensive equipment and is restricted to central testing laboratories. Aside from centrifugation, other plasma separation techniques (such as filtration) have been explored for POC use,^2–5^ but it remains a challenge to integrate plasma separation with downstream sample preparation steps (such as nucleic acid extraction).

Further, extraction of nucleic acids is often a critical step in sample preparation. Plasma contains substances (*e.g.* heme and immunoglobulins) that could inhibit amplification.^1^ Traditional RNA extraction uses silica columns or magnetic beads in a tube, and features a series of manual pipetting steps.^6^ A recent elegant strategy has been developed by several groups^7–12^ to perform nucleic-acid extraction in POC microfluidics, based on magnetofluidics and immiscible phases, sometimes referred to as IFAST (Immiscible Filtration Assisted by Surface Tension);^7,13–16^ specifically, nucleic acid-bound magnetic particles are transported through aqueous phases separated by immiscible oil or liquid wax, accomplishing some of the individual steps of an RNA extraction workflow (*i.e.* lysis, washing, and elution) within a single connected system without pipetting steps. However, there still remain important challenges. In assays where a whole-blood specimen is obtained at the POC (including finger-pricked blood tests which involve 100 μL or less of whole blood), plasma separation and nucleic acid extraction are still done as separate steps and with separate devices.^8,17,18^ For example, Ngo *et al.* 2023 (ref. 8) reports a POC HIV viral load quantification method from blood using a power-free plasma separation device and a separate magnetofluidic cartridge. Multiple liquid handling steps are required to transfer filtered plasma from device to a tube containing RNA extraction reagents to the magnetofluidic cartridge, adding complexity to the workflow. In addition, this method and others^11^ still require additional equipment such as micropipettes for sample transfer before the magnetofluidic steps and a laboratory shaking mixer for bead mixing and RNA capture prior to sample loading^8^ (ESI Table S1†).

An integrated, field-deployable device for sample preparation starting from whole blood and including nucleic acid extraction could increase access to testing and treatment for a variety of infectious diseases. A prime example of one such disease is hepatitis C virus (HCV), a leading cause of liver-related mortality worldwide and responsible for half of the annual liver cancer cases in the U.S.^19^ Encouragingly, recently developed direct acting antivirals can cure HCV in over 95% of people infected,^20^ but ∼40% of infected people in the U.S. are unaware of their infection.^21^ A major barrier lies in the two-step diagnostic process, requiring an initial antibody screen followed by an expensive RNA test to confirm active infection. Hence, availability of single-visit HCV tests could vastly improve detection and treatment. An ideal one-step diagnostic would be accurate, simple, rapid, affordable, and able to be performed by minimally trained users.^22^ RT-PCR assays are the gold standard test for HCV diagnosis, as they are sensitive, specific, and are robust across variants.^23^

**Fig. 1:**
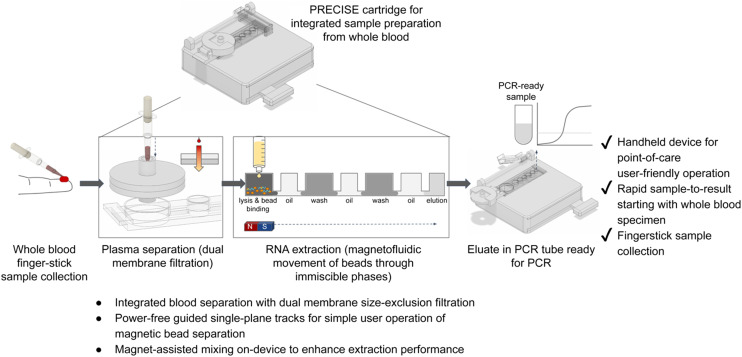
Design of PRECISE cartridge, which integrates plasma separation and RNA extraction for processing of whole blood at the point of care. Overview of workflow for blood sample preparation for HCV detection using the PRECISE cartridge. The PRECISE cartridge operates with as little as 50 μL of whole blood sample (which is compatible with fingerstick blood collection methods), drawn up by user with a tool that is then used to dispense the blood into the plasma separation component of the device. The dual-membrane filter separates out plasma from the sample, which is then withdrawn by user with a syringe from the filter to be dispensed into the extraction component of the device. Extraction is accomplished by using a magnetofluidic immiscible phase reagent separation approach to purify the RNA for PCR performed off-device. Overall, for the user, no pipetting steps are needed upstream of the cartridge (commonly used blood collection tools such as Minivette POCT (Sarstedt) or MICROSAFE tube (Drummond Scientific) fit right into our filter), the plasma separation step involves fluid handling with similar complexity to running LFA rapid tests, and the nucleic acid extraction requires the user to move two magnets either around a chamber to mix or along tracks to move the beads.

To overcome these limitations, we have developed PRECISE (P̲oint-of-care R̲NA/DNA E̲xtraction C̲artridge using I̲mmiscible phase S̲e̲paration), which leverages integration of plasma separation from whole blood with magnetofluidic immiscible phase RNA extraction for simple, power-free sample preparation for bloodborne pathogen detection (Fig. 1). This device features a dual-membrane size exclusion filter (containing glass fiber membranes for separating plasma from whole blood) integrated with a magnetofluidic nucleic acid extraction module, requiring only a syringe accessory for operation. The PRECISE cartridge aims to simplify sample preparation for whole blood specimens, to improve feasibility for POC high-performing PCR or RT-PCR diagnostic testing, as demonstrated in this work for detection of HCV.

## Results

### Dual-membrane size-exclusion filtration for plasma separation from whole blood

**Fig. 2:**
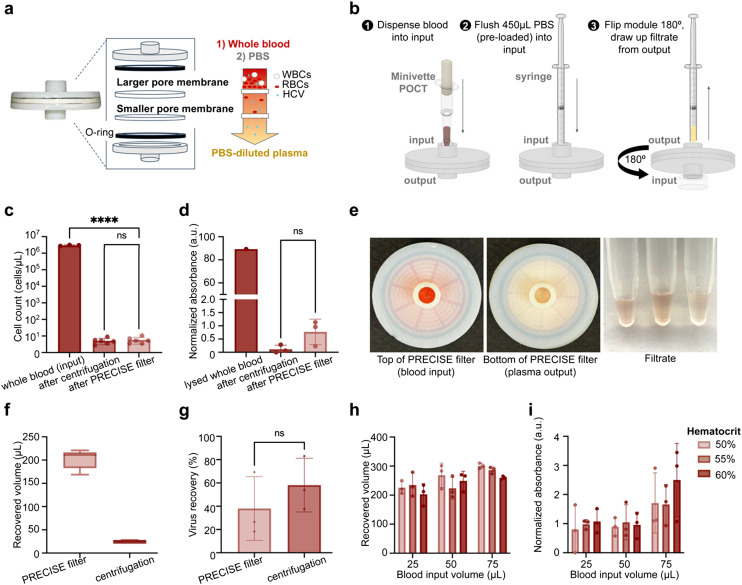
Design and characterization of dual-membrane size-exclusion filter for separating plasma from whole blood. **(a)** The filter allows for filtration of plasma from blood. The filter is constructed with an input and output piece, O-rings incorporated internally on each end, and a larger pore membrane (2.7 μm, glass fiber) followed by a smaller pore membrane (0.7 μm, glass fiber). The larger pore membrane functions to block larger blood components (such as white blood cells and some red blood cells), while the smaller pore membrane blocks the remaining cells and allows plasma and any present virus particles to pass through. **(b)** Workflow of plasma separation. The user dispenses the blood sample (*e.g.* collected from a Minivette, as demonstrated in this study) into the filter, flushes 450 μL of PBS (that was pre-loaded in a syringe) into the filter, and flips the filter upside down and draws the filtrate from the upward-facing outlet (see Fig. 4a for how the flipped filter looks in relation to the rest of the PRECISE cartridge.). **(c)** Plasma separation performance of the filter demonstrates its ability to separate the majority of cells from the blood sample with no significant difference from lab-based centrifugation. **(d)** Filtration causes minimal hemolysis of red blood cells, with no significant difference from centrifugation control. Hemolysis measured *via* hemoglobin absorbance peak at 414 nm peak and compared to fully lysed whole blood. **(e)** Images of filter after filtering blood. The red color at input (left) from blood sample on the membrane contrasts with the yellow color at output (middle) where only PBS-diluted plasma filtrate exits the filter, indicating successful retention of blood cells in the membranes. Images of the filtrate (right) are included, for three replicates after processing of 50 μL of whole blood. **(f)** Comparison of volume recovery of filter and centrifugation methods of plasma separation. The filter produces 201.8 ± 18.75 μL filtrate after input of 50 μL blood and 450 μL PBS, and centrifugation of 50 μL blood produces 24.6 ± 3.35 μL plasma. **(g)** Virus recovery from plasma separated from blood, spiked with whole inactivated HCV virus at 6.77 × 10^3^ IU mL^−1^ final concentration in blood, using filter and traditional centrifugation methods, extracted *via* benchtop RNA extraction protocol and amplified *via* RT-PCR. Recovery was calculated by comparing *C*_t_ values to RNA standard curve and demonstrated no significant difference between filter and centrifugation separation methods. **(h)** Recovered volumes of filtrate across hematocrit levels (50%, 55%, and 60%) and blood sample input volumes (25, 50, and 75 μL). There was no significant difference in recovered volumes across hematocrit levels. There was an observed trend in higher recovered volume with higher blood input, with significantly higher filtrate volume for 75 μL blood *vs.* 25 μL irrespective of hematocrit (*p* = 0.0012). **(i)** Hemolysis level in filtrate across hematocrit levels and blood sample input volumes, measured at absorbance peak 414 nm for hemoglobin. Within blood sample input volumes, there was no significant difference in hemolysis between hematocrit levels. There was a significant difference between 25 μL and 75 μL (*p* = 0.0219) and 50 μL and 75 μL (*p* = 0.0237) irrespective of hematocrit level, but there was no significant difference in hemolysis between 25 μL and 50 μL groups. Error bars in all figures indicate mean ± standard deviation. ****p < 0.0001, ***p < 0.001, **p < 0.01, *p < 0.05.

To achieve simple, low-cost plasma filtration, we developed a simple blood filtration strategy able to be interfaced with a microfluidic chip (Fig. 2a). The dual-layer 3D-printed filter consists of parallel glass fiber membranes of subsequently smaller pore sizes. Healthy red blood cells range from about 7–9 μm in diameter, so filtration using subsequent membranes of smaller pore sizes down to <1 μm effectively filters red blood cells (RBCs), white blood cells (up to 20 μm) and platelets (3–4 μm), while retaining the constituents of plasma (*i.e.* water, coagulants, proteins, viruses, and nucleic acids).^24^

For compatibility with point-of-care diagnostic use cases, using small volumes of fingerstick capillary blood, we aimed to accommodate up to 50 μL of blood. This volume was chosen to maximize assay sensitivity and ensure practicality with fingerstick blood collection, as collection of larger volumes (≥100 μL) is difficult with a single fingerstick.^25^ Further, the filter was designed to be compatible with capillary commercially available blood collection and dispensing tools (including ones sized to collect 50 μL, such as the Minivette POCT (Sarstedt)) to simplify the workflow and improve reproducibility. The user collects a capillary blood sample using the Minivette tool and dispenses the sample into the inlet of the filter. The user then dispenses PBS into the filter using a pre-filled syringe to push plasma through both membranes while retaining blood cells. To maximize recovered plasma volume, the inlet is then plugged, the filter is flipped, and the same syringe is attached to the outlet to draw filtered plasma out of the membranes with negative pressure. The filtered plasma in the syringe is then directly dispensed into the microfluidic cartridge.

The primary advantage of the dual-layer membrane approach is stringent filtration of blood components while limiting membrane clogging, maximizing the blood volume that is able to be filtered. We explored using only a single membrane for filtration, but found that filtration of 50 μL of blood with only a 2.7 μm glass fiber membrane resulted in inefficient filtration and red blood cell breakthrough, likely due to red blood cells' ability to deform to minimize resistance to flow^26^ (ESI† Fig. S2a). Using only the 0.7 μm glass fiber membrane led to improved filtration but resulted in membrane clogging, leading to significant hemolysis (ESI† Fig. S2a). Hemolysis is undesirable, as the release of heme from lysed RBCs is known to cause PCR inhibition.^27^ Overall, we found that glass fiber membranes with a pore size of 2.7 μm followed by 0.7 μm enabled efficient filtration while minimizing clogging and hemolysis (ESI† Fig. S2a and b), and used this combination in subsequent testing.

Glass fiber exhibited high liquid absorption, enabling high recovery of plasma when applying negative pressure. Other reported methods for membrane-based plasma filtration typically utilize polycarbonate track-etched membranes^2^ or a commercially-available asymmetric polysulfone membrane, the Vivid plasma separation membrane (Cytiva).^28–31^ We explored using polycarbonate membranes (of 3 μm and 0.4 μm pore sizes) due to their more consistent pore sizes and densities, but observed a higher degree of hemolysis compared to the dual glass fiber design (ESI† Fig. S2a). We also observed decreased volume recovery when using two polycarbonate membranes, likely due to low liquid absorption properties of polycarbonate (ESI† Fig. S2c). We also evaluated the asymmetric Vivid plasma separation membrane, used by several other membrane-based blood filtration approaches, with our filter design, and observed inefficient filtration and red blood cell breakthrough (ESI† Fig. S2b) compared to dual glass fiber membranes.

Using the dual glass fiber design we demonstrated high blood cell filtration efficiency (>99.9%) with 50 μL venous whole blood. Filtered plasma yielded an average cell count of 5.82 cells per μL, with no significant difference between centrifugation (5.13 cells per μL, *p* > 0.999) (Fig. 2c). This design also minimizes hemolysis (Fig. 2d) with no significant difference from gold-standard centrifugation (*p* = 0.0858). Visually, filtrate at the output was slightly pale yellow in color, as expected for diluted plasma, while deep red at the input, indicative of trapped RBCs (Fig. 2e). The filter yielded an average volume of 201.8 μL (s.d. = 18.75 μL) of diluted plasma across 9 replicates when 450 μL of PBS was added, resulting in an overall volume recovery of 40.4% (Fig. 2f).

### Virus recovery with plasma filter

To validate that the filter enables viruses to transport across the glass fiber membranes, we evaluated the recovery of inactivated HCV (whole virus) in spiked whole blood samples compared to gold-standard centrifugation. 50 μL of HCV-spiked whole blood at a concentration of 6.77 × 10^3^ IU mL^−1^ was separated using the filter (*n* = 3) and gold-standard centrifugation (*n* = 3). HCV RNA was extracted from filtered plasma using conventional benchtop RNA extraction and quantified by RT-PCR. Average recovery of HCV in the filter was 38.0% ± 27.4%, compared to 58.1% ± 23.1% from the centrifugation control (Fig. 2g). There was no significant difference in HCV recovery from the filter compared to gold-standard centrifugation (*p* = 0.3853).

### Evaluation of plasma separation across hematocrit levels and blood sample input volumes

Further, we sought to assess the capacity of the filter at a range of blood volumes and at various hematocrit levels to investigate risk of clogging or lysis at higher levels. It is widely known that blood obtained from fingerstick can have higher than normal hematocrit levels, due to capillary action in smaller capillaries causing greater water retention than venous collection.^32^ We prepared blood samples at a range of volumes (25 μL, 50 μL, and 75 μL) and blood hematocrit levels (50%, 55%, and 60%, *i.e.* at and beyond the high end of physiological levels) by adding or removing plasma. Blood samples were filtered with the PRECISE filter and filtrate was collected.

The filter was able to recover large volumes of plasma at all volumes and hematocrit levels tested, with an expected trend of higher plasma recovery for higher blood volumes (Fig. 2h). However, we also observed a correlation between the degree of hemolysis and input blood volume, both visually and quantitatively (ESI† Fig. S2 and 2i). There was significantly higher hemolysis (indicated by absorbance at 414 nm) between the 75 μL input volume group and 25 and 50 μL groups (*p* = 0.0219 and *p* = 0.0237, respectively), while there was no statistically significant difference between 25 and 50 μL (*p* = 0.9992) (Fig. 2i). We observed a trend of increasing hemolysis with increasing hematocrit within the 75 μL group only, but these differences were not statistically significant (*p* = 0.9963, 0.3903, and 0.3491). We determined that the filter can process up to 50 μL of whole blood at least up to 60% hematocrit without significant increases in hemolysis, which is beyond the expected physiological range (Fig. 2i).

### Fabrication of magnetofluidic nucleic acid extraction module

**Fig. 3:**
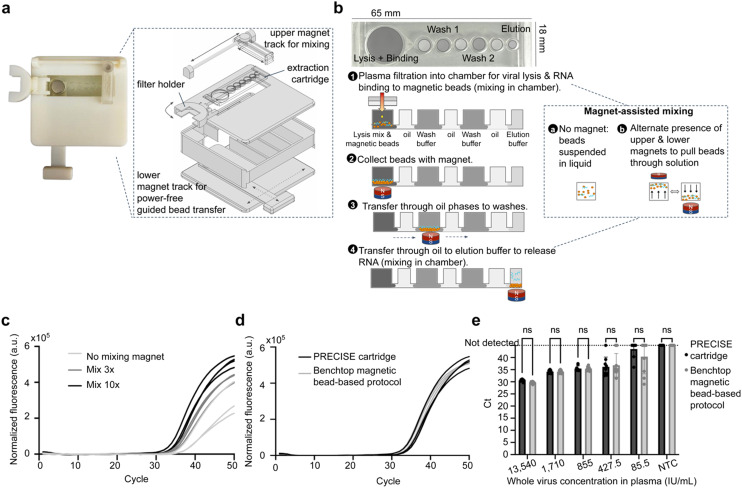
Design and characterization of nucleic acid extraction module. **(a)** Image (left) and schematic (right) of PRECISE device design. Device includes a magnet with a lower track for manually operated, power-free guided transfer of magnetic beads across the microfluidic extraction module. An upper magnet with a track facilitates mixing (see Fig. 3b). A holder for the filter sits to the left of the extraction module for movement of filter above and away from the first chamber of module for sample addition when whole blood starting samples are used, as in Fig. 2 and 4. **(b)** Design of magnetofluidic immiscible-phase extraction module (65 × 18 mm). The module includes an initial large chamber to accommodate appropriate lysis/binding buffer mix and 200 μL sample input, two wash chambers with wash buffer, and an elution chamber with elution buffer, all separated by chambers for mineral oil. Chambers are pre-loaded with reagents, and the extraction process functions as follows: 1) plasma sample is added (*via* dual-membrane filtration as in Fig. 2, or conventionally separated plasma as is used in this figure) to the first chamber containing lysis buffer, purification buffer, proteinase K, and magnetic beads functionalized for binding nucleic acids. Magnet-assisted mixing is performed by alternating presence of upper and lower magnets above and below the chamber, respectively, to draw beads through solution. Beads are then collected with the lower magnet and transferred through the oil chambers into the subsequent washes and finally into the elution chamber, where the magnet mixing is performed again before removal of eluate from the final chamber. **(c)** Use of the upper magnet for magnet-assisted mixing improves RNA extraction performance on the module, as demonstrated by the differences in RT-PCR amplification between no magnet-assisted mixing, mixing 3 times, and mixing 10 times in the lysis/binding and elution steps. **(d)** Representative RT-PCR curves for HCV RNA extraction on PRECISE cartridge and with benchtop magnetic bead extraction kit, from whole, inactivated HCV spiked into 25 μL of DNA-cleared plasma (estimated volume of plasma from 50 μL blood sample) and diluted by PBS to 200 μL to represent the estimated sample volumes determined from Fig. 2f, shown at 6770 IU mL^−1^ starting concentration in plasma sample. **(e)** RT-PCR amplification at varying concentrations of whole, inactivated HCV spiked into 25 μL of DNA-cleared plasma and diluted by PBS to 200 μL. All replicates amplified for both cartridge and benchtop comparison at 855 IU mL^−1^, with no significant differences between extraction methods for any concentration. Error bars in all figures indicate mean ± standard deviation. ****p < 0.0001, ***p < 0.001, **p < 0.01, *p < 0.05.

To implement immiscible-phase nucleic acid extraction, we designed a microfluidic module with circular chambers for each step of extraction (lysis/binding, two washes, elution) connected by chambers containing mineral oil (Fig. 3b, ESI† Fig. S4a). This design allowed magnetic beads to move freely, controlled by a permanent magnet, between chambers of different nucleic acid extraction reagents separated by oil barriers. The chambers were left open at the top for simple reagent loading prior to experiments, but future iterations of the system can implement enclosed chambers with point-of-care friendly reagent loading mechanisms. The module was 3D printed and sealed with a Poly(methyl methacrylate) (PMMA) base attached with glue to create the bottom surface, as the magnetic bead movement was found to perform best with a bottom surface that was hydrophobic with low surface roughness. Modules with PMMA base demonstrated significantly improved RNA extraction performance with less variability compared to modules with a 3D printed resin base (ESI† Fig. S5a), likely due to variable surface roughness during DLP 3D printing and post-processing.

The module was designed to hold at least 200 μL of sample in the lysis/binding chamber in addition to the necessary reagents without disturbing the oil barriers separating reagents in subsequent chambers. We found that having a small interface between the oil and buffer phases (*i.e.* 0.5 mm × 0.25 mm) resulted in effective separation across a wide range of sample volume inputs, which is consistent with a small Bond number in this geometry such that a relatively high surface tension (over gravity) results in stable phase separation. In addition, we found chambers accommodating larger volumes of oil maintained the oil barrier efficiently (ESI† Fig. S4b). Circular reagent chamber geometries were chosen as sharp corners can trap magnetic beads or create air pockets that disrupt connection across immiscible phases,^33^ whereas the triangular narrowing design between reagent and oil chambers helped to guide the beads towards the centerline to cross into subsequent chambers (ESI† Fig. S4a).

We then further investigated the chosen design's ability to hold a wide range of sample volumes without disturbing the oil barriers separating extraction reagents. Using this design, the module was able to hold up to 300 μL of sample with no disturbance to the oil barrier (ESI† Fig. S4c) and up to 350 μL with minimal disturbance, which is well beyond the range of volumes recovered by the filter at a 50 μL blood input (Fig. 2e and h). Further, up to 450 μL could be added without reagents leaking into subsequent reagent chambers.

A device was designed to hold the microfluidic extraction module above a lower magnetic track containing a neodymium magnet manually controlled by the user for movement in four cardinal directions on one plane (Fig. 3a). An upper magnet on a track controlled manually by the user sat above the module for magnet-assisted mixing: moving the upper magnet above the chamber pulled the magnetic beads through the liquid, then moving the upper magnet away and replacing the lower magnet below the chamber pulled the beads back to the bottom surface (Fig. 3b, inset). The use of the upper magnet to stimulate mixing was found to greatly improve extraction performance, likely due to improved RNA binding to magnetic beads (Fig. 3c).

### Characterization of immiscible-phase magnetofluidic RNA extraction with spiked hepatitis-C virus in human plasma

The PRECISE device uses a magnetofluidic immiscible-phase reagent separation approach to nucleic acid extraction; thus, the RNA extraction chemistry is incompatible with alcohol-based washing common in many traditional magnetic extraction kits, as ethanol's low surface tension was found to cause issues maintaining phase barriers. Further, residual ethanol remaining on magnetic beads during elution can inhibit PCR (bead drying is not feasible in an enclosed system such as PRECISE).^34^ Thus, a pH-induced nucleic acid binding chemistry from a commercial nucleic acid extraction kit was selected to avoid alcohol-based washing.

Extraction performance was first tested with a starting sample of inactivated HCV spiked into human plasma. The plasma sample was pipetted into the module (pre-filled with reagents) *via* the first microfluidic chamber for viral lysis and binding of RNA to the magnetic beads; after an initial 3 minute incubation, the chamber was mixed by alternating presence of upper and lower magnets above and below the chamber, followed by a 2 minute incubation. The lower magnet, guided along the track by the user, pulled the beads from the lysis/binding chamber through the first oil chamber into the first wash, through the second oil chamber into the second wash, and through the third oil chamber into the elution chamber. Another 3 minute incubation, mixing with upper and lower magnets, and 2 minute incubation was performed to allow the RNA to detach from the beads. Finally, the lower magnet was used to hold the magnetic beads to the edge of the chamber and the eluted RNA was removed for PCR amplification. The total extraction can be completed in about 14 minutes, which includes 5 minutes of incubation each in the first and last chambers, time for mixing in first and last chambers, and time for careful transfer to minimize bead loss (ESI† Fig. S3 and Video S1).

In order to improve RNA extraction performance with the module, we aimed to 1) minimize magnetic bead loss between chambers, 2) maximize RNA binding and elution efficiency, and 3) ensure removal of all PCR inhibitors. We performed multiple RNA extraction experiments using inactivated HCV spiked in human plasma to characterize the effect of several parameters in achieving these three goals.

First, the magnetofluidic approach utilized by the PRECISE system relies upon efficient transfer of magnetic beads between reagents. If there is excess magnetic bead loss between the lysis/binding and elution chambers, there will be significant sample loss resulting in poor RNA extraction efficiency. To ensure minimal magnetic bead loss, cylindrical neodymium magnets of various sizes were evaluated, including a 12.7 mm diameter × 12.7 mm height magnet and a 6.4 mm × 3 mm magnet. It was determined both visually and quantitatively that the larger 12.7 mm diameter magnet resulted in less bead loss and higher extraction efficiency (ESI† Fig. S5b). While the 12.7 mm diameter magnet requires a slightly larger track design than the other magnets, thus increasing the overall footprint of the PRECISE device (though not beyond a handheld size), it has the advantage of being able to recover trapped beads in the oil phases from further away due to its stronger magnetic field, resulting in a cleaner transfer that ultimately allows for a smoother and faster workflow.

Next, we sought to optimize the number of mixing steps (resuspension of the magnetic beads in the chamber with the upper magnet) in the lysis/binding chamber and elution chamber to maximize RNA binding and elution efficiency. Implementation of efficient on-cartridge mixing is essential for point-of-care sample preparation, as a major limitation of previously reported methods is the necessity of mixing the sample with a vortex mixer or incubating the sample on a shaking/rotating mixer prior to RNA extraction, which is a barrier to integration and adds cost and complexity to the workflow. We performed on-cartridge RNA extraction with a various number of mixing steps with the upper magnet in the lysis/binding chamber and elution chambers. Performing more mixing steps greatly improved RNA extraction efficiency; resuspending the beads with the upper magnet 10 times resulted in earlier qPCR amplification of extracted RNA than three mixing steps and no mixing (Fig. 3c). Based on these results, we selected 10 mixing steps to implement in the workflow to maximize RNA binding and elution.

Further, we aimed to ensure elimination of potential PCR inhibitors by optimizing the washing step. In nucleic extraction, the washing buffer ensures that all unbound contaminants in the sample, such as proteins and salts, are removed, while the nucleic acids stay bound to magnetic beads. It has been previously reported that immiscible phase nucleic acid purification can eliminate PCR inhibitors through transfer of magnetic particles through the oil/hydrophobic liquid,^7^ eliminating the need for transfer through a wash buffer. To explore the possibility of eliminating wash steps, we performed on-cartridge RNA extraction using cartridges with only one wash chamber, compared to our original cartridge design with two washes, and benchtop extraction. Although there was no statistically significant difference in C_t_, we observed more variability in *C*_t_ in cartridges with only one wash (S.D. of 2.94, compared to 0.386 with two washes), potentially due to inconsistent washing and presence of inhibitors (ESI† Fig. S3d). We then explored the potential of implementing mixing within each wash chamber to further improve removal of PCR inhibitors. However, we found that performing mixing in the wash chamber actually reduced RNA extraction efficiency (ESI† Fig. S3e). It is possible that, because RNA binding to the functionalized magnetic beads relies upon acidic pH (<5.0), and the wash buffer is less acidic than the binding buffer, too much agitation of the magnetic beads within the wash chambers results in unwanted removal of RNA from the magnetic beads.

### Sample-to-result demonstration of integrated PRECISE cartridge using spiked hepatitis-C virus in human whole blood

**Fig. 4:**
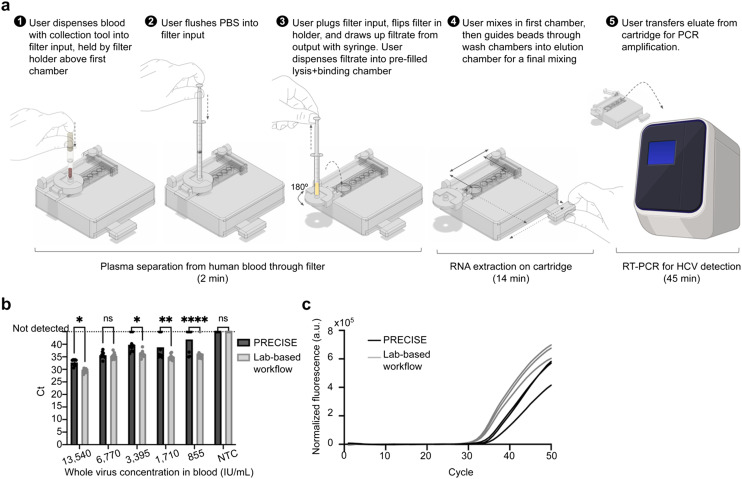
Demonstration of PRECISE device for sample-to-answer HCV detection. **(a)** Integrated workflow for PRECISE device for preparing whole blood sample for HCV detection *via* RT-PCR involves a plasma separation step *via* plasma filter and an RNA extraction step *via* a magnetofluidic immiscible-phase cartridge manually operated with a guided magnet track. 50 μL of fingerstick blood collected with a blood collection tool is dispensed into the inlet of filter, followed by 450 μL PBS flushed with a syringe. The filter is flipped and the syringe is used to draw out filtrate from the outlet, then dispense into first chamber of extraction cartridge. The user performs on-cartridge extraction by guiding a magnet to move RNA-bound functionalized magnetic beads through chambers containing lysis/binding buffers, wash buffers, and finally elution buffer all in connected sequence and separated by mineral oil. The user then transfers the eluate from the final chamber to a PCR plate for RT-PCR on a QuantStudio 3 instrument. **(b)** Detection of varying concentrations of whole, inactivated HCV spiked into 50 μL blood when processed with lab-based workflow (centrifugation for plasma separation, benchtop magnetic bead protocol for RNA extraction) and PRECISE device (integrated blood filter for plasma separation and magnetofluidic immiscible-phase cartridge for RNA extraction). Extracted samples from both approaches were amplified for detection *via* RT-PCR on QuantStudio 3 system. All replicates (*n* = 3, each technical replicate run in triplicate on PCR plate) for PRECISE device and lab-based workflow amplified at 6770 IU mL^−1^ with no significant difference between workflows. **(c)** Real-time fluorescence curves of RT-PCR comparing lab-based workflow and PRECISE device for sample preparation from whole blood at 13 540 IU mL^−1^. Error bars in all figures indicate mean ± standard deviation. ****p < 0.0001, ***p < 0.001, **p < 0.01, *p < 0.05.

Finally, we integrated the plasma separation and RNA extraction into a singular, continuous workflow of blood sample preparation for nucleic acid detection in point-of-care settings (Fig. 4a and ESI† Video S1). We incorporated a filter holder into the PRECISE device to position the filter above the microfluidic extraction module on the magnetic track (Fig. 3a and 4a). The holder allows for secure positioning and movement of the filter above and away from the extraction module as needed, holding it with inlet facing upward for sample and PBS dispensing and flipping it to face with outlet upward for filtrate withdrawal. 50 μL of blood was first collected from a droplet with a capillary collection tool (in point-of-care applications, this would be collected directly from a fingerstick puncture, but for this work's purpose, it was collected from a bead of blood formed from a pipette to simulate fingerstick collection). The collection tool was then used to add the blood directly to the top of the filter by pushing the plunger of the tool. PBS was added *via* syringe to flush the sample through the membranes. The filter was then plugged, flipped 180° on the holder, and the syringe was used to draw out any remaining plasma filtrate. The filtrate was directly dispensed into the lysis/binding chamber.

The extraction workflow described above was then performed using the magnet (ESI† Video S1). The timing for the entire integrated sample preparation workflow was approximately 16 minutes: 2 minutes for plasma separation, and 14 minutes for RNA extraction as described above. As a standard comparison, a lab-based workflow that included centrifugation for plasma separation from whole blood and the benchtop extraction protocol was also performed. Using spiked inactivated HCV at a range of concentrations, PRECISE demonstrated a limit of detection of 6770 IU mL^−1^ in 50 μL of whole blood (Fig. 4b), with slightly delayed PCR amplification (∼3 cycles) compared to gold-standard laboratory sample preparation (Fig. 4c).

## Discussion

PRECISE fully integrates plasma separation and nucleic acid extraction into one device, enabling streamlined sample preparation from whole blood. Compared to previous sample preparation methods, PRECISE offers several advantages, as it operates on whole blood (not previously separated plasma or serum) as the starting sample, including small volumes (equivalent to those obtained *via* finger prick). Operation of the cartridge requires no additional equipment (other than a syringe pre-loaded with buffer) or electricity. For the user, no pipetting steps are needed upstream of the cartridge (commonly used blood collection tools such as Minivette POCT (Sarstedt) or MICROSAFE tube (Drummond Scientific) fit into our filter), the plasma separation step involves fluid handling with similar complexity as running LFA rapid tests, and the nucleic acid extraction requires the user to move two magnets either around a chamber to mix or along tracks to move the beads.

The PRECISE cartridge features several technical innovations toward integrated sample preparation. The method features a novel dual-membrane plasma filter, which was integrated into the cartridge, compared to previous devices that required a separate plasma separation device with external sample transfer between.^8,17,18^ Nucleic acid extraction was built on a magnetofluidic immiscible filtration approach previously developed by other groups;^7,9,11,12^ in contrast with previous manual magnetofluidic extraction devices, our design added a guided magnetic track for ease of use and reproducibility (for magnetic bead transfer through the reagents), and an upper magnet to resuspend magnetic beads and facilitate mixing to attain high nucleic acid-binding efficiency (whereas many previous methods required a separate shaking, vortex, or rotating mixer to perform the binding step^8,11^). We observed that smaller interfaces and larger oil volumes maintained a stable configuration involving immiscible phases; this observation may arise from the balance of interfacial tension and capillary forces,^35^ which can be explored in the future through additional simulations.

This study takes a step towards improved POC HCV detection. We combined the PRECISE sample preparation with conventional PCR amplification, starting with 50 μL of whole blood. This study demonstrated a limit of detection (LoD) of 6770 IU mL^−1^ of inactivated HCV virions spiked into whole blood, which is close to but still higher than the minimal requirements for HCV diagnostics in decentralized settings (*i.e.* <3000 IU mL^−1^, as described by the FIND target product profile^22^). However, we note that starting with 50 μL of plasma starting sample, the PRECISE cartridge alone demonstrates a LoD of 855 IU mL^−1^. Based on this data, and the data shown on the efficacy of the plasma filtration alone, we hypothesize that some of the volume from 50 μL was lost in the filter, and hence future work can focus on reducing the dead volume lost, or starting with larger volumes (than 50 μL) of whole blood collected at the POC^36,37^ would likely lower the LoD. In terms of time to result, the PRECISE technique currently requires 16 minutes. As our group has previously published a rapid plasmonic PCR platform that can complete 45 cycles in less than 15 minutes,^38^ sample-to-answer detection from fingerstick blood in approximately 30 minutes would be significantly faster than the 60 minutes of Cepheid GeneXpert, which is the only FDA-approved POC HCV RT-PCR test using whole blood as the starting sample.^39^

## Materials and methods

### Fabrication of plasma filter for dual-membrane size-exclusion filtration

Filters were designed using AutoCAD Fusion360 and 3D printed using VeroWhite resin on a Stratasys Objet 30 Pro. Filters were composed of two identical printed pieces: a syringe inlet port piece and an outlet piece. Each piece included a ringed spoke design printed on the surface in contact with the membranes to help liquid distribute evenly across the membranes. Square-profile 20 mm ID O-rings (1171N113, McMaster-Carr) were inserted into the syringe inlet port and outlet layers. 2.7 μm and 0.7 μm pore size glass fiber membranes (APFD02500 and APFF04700, Millipore) were cut to a diameter of 25 mm using a leather hole puncher. The membranes were stacked with the 2.7 μm pore size on top facing the syringe port and the 0.7 μm membrane pore size at the outlet piece. The pieces were assembled with edges sealed using light-cure adhesive (Loctite 3525) and cured with 3000 flashes in a UV curing unit (Otoflash G171).

### Separation efficiency of plasma from blood

Healthy control human whole blood was purchased from HumanCells Biosciences; all specimens were de-identified and tested negative for HIV1/2, HBV, HCV and syphilis prior to receipt. Whole blood was stored at 4 °C and handled using BSL-2 facilities and protocols. For plasma separation using the filter, 50 μL of whole blood was inserted into the inlet port of the filter directly on top of the membrane using a pipette. A 1 mL Luer slip tip syringe pre-loaded with 450 μL of PBS was inserted into the inlet port, and the plunger was slowly pushed to push plasma through the membrane. The syringe was removed and the inlet was then sealed using a 3D-printed plug (PlasClear resin, Asiga). The filter was flipped over and the syringe was then placed into the outlet port and drawn upwards, drawing filtered plasma into the syringe using negative pressure. Once the plunger reached the end of the syringe, the filtered plasma was expelled either directly onto the first chamber of the extraction module or into a clean 1.5 mL tube.

Gold-standard controls were centrifuged at 1500 g for 10 minutes, and plasma supernatant was removed carefully using a pipette into a clean 1.5 mL tube. 10 μL of each filter and centrifugation control sample were then diluted 1 : 1 with Trypan Blue (final volume 20 μL) and added to an Invitrogen Countess cell counting slide (C10228, ThermoFisher Scientific). Input whole blood comparison samples were diluted 1000-fold prior to addition of Trypan Blue. Cell counts were obtained using an automated cell counter (Invitrogen Countess 3, ThermoFisher Scientific).

### Determination of hemolysis from plasma separation

The level of hemolysis was determined by measuring absorbance of each sample separated using the filter and gold-standard centrifugation at 414 nm, which is a known absorbance peak of hemoglobin^40^ using a NanoDrop One spectrophotometer (ThermoFisher Scientific). 2 μL of each sample was measured using the spectrophotometer, using PBS as a blank. Gold-standard centrifugation and fully lysed whole blood samples were diluted to the same volume as the filtered plasma samples using PBS and used as controls. The obtained absorbance values were baseline-corrected to achieve only positive values.

### Viral recovery with plasma filter

5 μL of inactivated HCV (NATHCV-0005, ZeptoMetrix) was spiked into 45 μL whole blood for a final concentration of 6.77 × 10^3^ IU mL^−1^. Spiked 50 μL whole blood samples were separated into plasma using the filter and gold-standard centrifugation. Resulting volumes of separated plasma were measured using a micropipette. HCV viral RNA was extracted from resulting plasma samples using a modified, optimized benchtop extraction protocol using the Invitrogen ChargeSwitch gDNA Mini Tissue Kit (ThermoFisher Scientific). RNA was quantified *via* RT-qPCR through generation of a standard curve using a range of concentrations of HCV quantitative synthetic RNA (VR-3233SD, ATCC). Virus recovery in separated plasma was back-calculated using the quantified concentration of eluted RNA with the assumption of 50% RNA extraction efficiency.

### Evaluation of plasma separation across range of hematocrit levels and blood sample input volumes

To evaluate the filter at a range of blood volumes and various hematocrit levels, we prepared blood samples at artificial blood hematocrit levels (50%, 55%, and 60%) by centrifuging whole blood at 1500 × *g* for 10 minutes, removing the separated plasma to calculate the leftover volume of packed blood cells (while hematocrit refers to red blood cells percentage only, this is a close estimate, as the packed blood cells are >99% RBC).^41,42^ We then added plasma back at appropriate volumes to generate the correct total% RBC in each sample. Different volumes of the artificially-created hematocrit blood were added (25, 50, and 75 μL) to filters and filtrate was collected for analysis. Hemolysis and cell count of filtrate were tested as described above.

### Fabrication of magnetofluidic nucleic acid extraction module

The module was 3D-printed with open chambers using PlasClear resin (Asiga) in an Asiga Max X 3D printer, cleaned with 100% isopropyl alcohol, and cured with 2000 flashes in the UV curing unit. The chambers were sealed to a sheet of acrylic (PMMA) measuring 60 × 15 × 0.25 mm using light-cure adhesive and cured using 2000 flashes in the UV curing unit. The overall module was 65 mm × 18 mm × 3.25 mm, with raised edges on both ends of the long side to hold the module in the designated space above the magnetic track. The chambers were designed as cylindrical for easy reagent filling; the lysis chamber had a diameter of 15 mm and height of 3.25 mm to hold a maximum of approximately 575 μL of liquid. The wash chambers had diameters of 6 mm and heights of 3.25 mm to hold a maximum of approximately 90 μL of liquid. The elution chamber had a diameter of 4 mm and height of 3.25 mm to hold a maximum of approximately 40 μL of liquid. Chambers for oil connected the chambers (sized 7 mm diameter for the oil after the larger lysis chamber, and 5 mm for the other two oil chambers), with triangular transitions between all chambers to aid in guiding beads for transfer (length of 3 mm between lysis chamber and first oil chamber, and 1 mm between all other chambers). All dimensions are given in ESI† Fig. S4. Reagents and oil could be pre-loaded directly into chambers from above *via* pipette.

### Fabrication of magnetic track for holding extraction module

The magnetic track was 3D-printed using VeroWhite Plus resin (Stratasys) in a Stratasys Objet 30 Pro 3D printer. Printed components were post-processed with an alkaline cleaning bath and dried in an oven at 40 °C. The lower track on which the extraction module sits was designed to guide a cylindrical 12.7 × 12.7 mm neodymium magnet (D88-N52, K&J Magnetics) in four cardinal directions on one plane, held by a 3D-printed arm and manually controlled by the user. The extraction module was held in a slot on the lid of the magnetic track to position it above the lower track. The upper track to hold a magnet for mixing the magnetic beads in the module chambers was designed to guide a cylindrical 6.4 mm × 3 mm neodymium magnet (D42-N52, K&J Magnetics) from left to right on one axis above the module chambers; it could also be flipped backward away from the module by rotating in place on the axis. A filter holder was designed into which the filter could be slotted and removed as needed, for use with experiments involving whole blood starting samples. The holder positioned the filter's outlet above the first chamber of the extraction module for initial blood sample deposit and PBS flush into inlet facing upward, to direct any early filtrate droplets that may come through the outlet before withdrawal *via* syringe. The holder could be flipped 180°, as needed for filtrate withdrawal, while still holding the filter securely and positioning the filter, now facing with outlet port upward, outside of the track away from the extraction module to allow for filtrate withdrawal *via* syringe.

### HCV RNA extraction with benchtop protocol

The ChargeSwitch gDNA Mini Tissue kit (ThermoFisher Scientific) was selected for extraction, as it utilizes pH-induced nucleic acid binding and does not require alcohol-based washing. The ChargeSwitch protocol was modified and optimized for reduced reaction volumes. First, a lysis and binding mix was prepared, consisting of 140 μL of lysis buffer, 20 μL of proteinase K (20 μg mL^−1^), 50 μL N5 purification buffer, and 8 μL of ChargeSwitch magnetic beads per reaction for a 200 μL filtered plasma input. These volumes were modified slightly to scale with different sample volumes, as needed (*i.e.* for undiluted centrifuged plasma samples with an expected volume of 25 μL, the reaction consists of 17.5 μL of lysis buffer and 6.25 μL of purification buffer, with the same volumes of proteinase K and magnetic beads). The mixture was added to each plasma sample in a 1.5 mL tube, gently pipette mixed ten times, and allowed to incubate at room temperature for 5 minutes. The samples were placed on a 1.5 mL magnetic rack (New England Biolabs) until the beads were separated out of solution, and the supernatant was removed using a pipette. The beads were washed twice with 200 μL of wash buffer (40 μL for undiluted centrifuged plasma samples). 30 μL of elution buffer was added and the beads were pipette mixed ten times to resuspend. After a 5-minute incubation at room temperature, the beads were once again separated using the magnetic rack, and the eluted RNA was transferred to a clean PCR tube for further processing. The same reagent volumes were used in the magnetofluidic extraction module for a 200 μL plasma input, with the exception of 90 μL of wash buffer used in the wash chambers instead of 200 μL.

### Immiscible-phase magnetofluidic RNA extraction with whole hepatitis-C virus in human plasma

Initial characterization of the RNA extraction module (optimizing magnet size, mixing, and washing steps) was performed by spiking inactivated HCV into DNA-cleared plasma for a final concentration of 6.77 × 10^3^ IU mL^−1^. The spiked plasma sample was added to the lysis/binding chamber and incubated for 3 minutes. The beads were then mixed by alternating the mixing magnet on top of the chamber with the extraction magnet underneath the chamber 10 times. Beads were then collected at the bottom of the chamber and transferred horizontally through the oil phase into each wash chamber before transfer to the elution chamber. The beads incubated in the elution chamber for 3 minutes. The beads were mixed ten times and resuspended using the mixing magnet for an additional 2-minute incubation. The extraction magnet was used to separate the beads from solution, and the 30 μL eluate was transferred to a clean PCR tube using a pipette.

To evaluate the limit of detection of magnetofluidic RNA extraction, serial dilutions of inactivated HCV in PBS were spiked into DNA-cleared plasma (System Biosciences) to obtain concentrations from 1.3 × 10^4^ IU mL^−1^ to 85.5 IU mL^−1^, with three replicates per concentration. Performance at each concentration was compared to the optimized benchtop extraction protocol.

### RT-qPCR quantification of extracted RNA

Previously published primers and probes designed from the conserved 5′ untranslated region (UTR) of the HCV genome were used to amplify HCV RNA (MAD-1 primer 5′-TGCTAGCCGAGTAGYGTTGG-3′, MAD-2 primer 5′-ACTCGCAAGCACCCTATCAG-3′, and MAD-3 probe 5′-ACCACAAGGCCTTTCGCGAC-3′).^43^ The RT-qPCR reaction mix consisted of PrimeScript One-Step RT-PCR master mix (1× final concentration), forward and reverse primers (500 nm), and probes (125 nm) with a final volume of 20 μL. RT-qPCR was performed using an Applied Biosystems QuantStudio 3 thermocycler (ThermoFisher Scientific) using the following cycling conditions: 52 °C for 5 minutes, 95 °C for 10 seconds, and 50 cycles of 95 °C for 5 seconds and 60 °C for 30 seconds. For quantification of extracted RNA concentration, a standard curve was generated using ten-fold serial dilutions of HCV quantitative synthetic RNA (ATCC, #VR-3233SD) from 8.3 × 10^7^ copies per mL to 830 copies per mL, with three technical replicates per concentration. A best fit equation generated from the plot of *C*_t_*vs.* log concentration was used to calculate PCR efficiency and estimate the concentration of extracted RNA based on the average *C*_t_ of three technical replicates.

### Integrated device and workflow for RNA extraction from whole hepatitis-C virus in human whole blood

To investigate limit of detection of the integrated system, five serial dilutions of inactivated HCV spiked into whole human blood were prepared as samples: 1.35 × 10^4^ IU mL^−1^, 6.77 × 10^3^ IU mL^−1^, 3.39 × 10^3^ IU mL^−1^, 1.71 × 10^3^ IU mL^−1^, and 8.55 × 10^2^ IU mL^−1^, as well as an no-template control with TE buffer spiked into whole human blood. Three experimental replicates of each concentration were performed (three cartridge extractions per concentration).

For separating plasma from the starting blood sample, the filter was placed in the holder of the PRECISE device to position the outlet directly above the first chamber of the extraction module. A pipette was used to generate a blood bead of 50 μL, simulating a fingerstick blood bead, then a Minivette POCT (Sarstedt) capillary collection tube was used to draw up the blood. The collection tool was then used to add the blood directly to the top of the filter by pressing down on the plunger. 450 μL of PBS was next flushed into the inlet port of the filter *via* 1 mL slip tip syringe. The inlet was plugged with a 3D printed plug, then the filter was flipped 180° with the filter holder to position it away from the extraction module with outlet port facing upward. The syringe, in a starting position with the plunger pressed all the way down, was then inserted into the outlet port and used to draw up filtrate from the filter. The syringe was then used to deposit the filtrate into the first chamber of the extraction module. The extraction workflow described previously was then performed using the magnets.

A lab-based workflow was performed as a standard comparison. Inactivated HCV was spiked into whole human blood, and the blood samples were centrifuged at 1500 × *g* for 10 minutes to separate the plasma. Separated plasma was pipetted off the top of the separated samples and used in the benchtop extraction protocol described previously.

RT-PCR was performed using eluted samples; each experimental replicate was run in triplicate in the PCR plate on the QuantStudio 3 system, according to RT-PCR protocol described previously.

## Statistics

All statistical analysis was performed with GraphPad Prism 10. All data were collected in triplicate (*n* = 3) unless otherwise specified. *t*-tests were used for comparisons between two groups. *Post hoc* multiple comparisons were performed when necessary using one-way ANOVA Tukey's multiple comparisons test, or two-way ANOVA Sidak's multiple comparisons test when multiple conditions were being compared at once.

### Live subject statement

All blood samples used were purchased as de-identified specimens from HumanCells Biosciences. All human blood samples were collected by HumanCells Biosciences from fully consented, IRB-approved donors who have tested negative for HIV1/2, HBV, HCV, and syphilis, under IRB #2023-0213 approved by Pearl IRB, in adherence with Good Clinical Practices outlined by the U.S. Food and Drug Administration.

## Data availability

The data supporting this article have been included as part of the ESI.†

## Author contributions

Conceptualization: AGA, CMV, SKS. Methodology: AGA, CMV, SKS. Investigation: AGA, CMV. Visualization: AGA, CMV. Funding acquisition: AGA, CMV, SKS. Project administration: AGA, CMV. Supervision: AGA, CMV, SKS. Writing – original draft: AGA, CMV. Writing – review & editing: AGA, CMV, SKS.

## Conflicts of interest

All authors declare that they have no competing interests.

## Supplementary Material

LC-024-D4LC00571F-s001

LC-024-D4LC00571F-s002
