# Supplementary material for: Integrated device for plasma separation and nucleic acid extraction from whole blood toward point-of-care detection of bloodborne pathogens
Source: Lab Chip. 2024 Oct 18;24(22):5124–36. doi: 10.1039/d4lc00571f (PMC11487500; doi:10.1039/d4lc00571f)
Supplement: LC-024-D4LC00571F-s001 [file LC-024-D4LC00571F-s001.pdf]

Supplementary Information (SI) for Lab on a Chip.
This journal is © The Royal Society of Chemistry 2024

SUPPLEMENTAL MATERIAL

Integrated device for plasma separation and nucleic acid extraction from whole blood toward point-of-care detection of bloodborne pathogens

Abigail G. Ayers,[†a] Christia M. Victoriano,[†a] Samuel K. Sia*[a]

[a]Department of Biomedical Engineering, Columbia University; New York, NY 10027, USA

*Corresponding author. Email: ss2735@columbia.edu

[†]These authors contributed equally to this text.

SUPPLEMENTAL TABLES

Supplemental Table 1. Comparison of work demonstrating whole-blood magnetofluidic sample processing for bloodborne viral disease detection. References indicated from main text.

Work	Bloodborne viral disease target	Power-free sample preparation	Integrated sample preparation from blood	Point-of-care-friendly sample collection & loading
Ngo et al., 2023 (ref. 8)	HIV	-<br>Arduino-controlled magnetic track	-<br>whole blood starting sample, a separate device for plasma separation; 3 min blood separation, ~27-min extraction, 15 min RT-PCR amplification on-device (total: ~45 min sample-to-result)	-<br>100 µL starting volume, requires pipetting
Berry et al., 2014 (ref. 14)	HIV	+<br>manual placement and movement of a magnet under device	-<br>serum starting sample, lysis/bead-binding done off-device; no blood separation, 5-10 min extraction + 50 min RT-PCR amplification off-device (total: ~1 hour sample-to-result)	-<br>100 to 500 µL starting volume, requires pipetting
Neto et al., 2017 (ref. 18)	HCV	-<br>computer-controlled magnetic track	-<br>plasma initial starting sample; blood separation off-chip, ~45 min nucleic acid extraction, ~72 min RT-PCR amplification on-device (total: ~117 min sample-to-result)	-<br>200 µL starting volume, requires pipetting
Ayers/Victoriano et al., 2024 *(this work)*	HCV	+<br>power-free fixed magnetic track in device, manipulated manually by user but with guided control	+<br>whole blood starting sample, plasma separation from blood on-device; 2 min blood separation, 14 min extraction, 45 min RT-PCR (total: ~1 hour sample-to-result)	+<br>50 µL fingerstick collection, Minivette POCT tool acts as sample collector and dispenser into device

SUPPLEMENTAL FIGURES

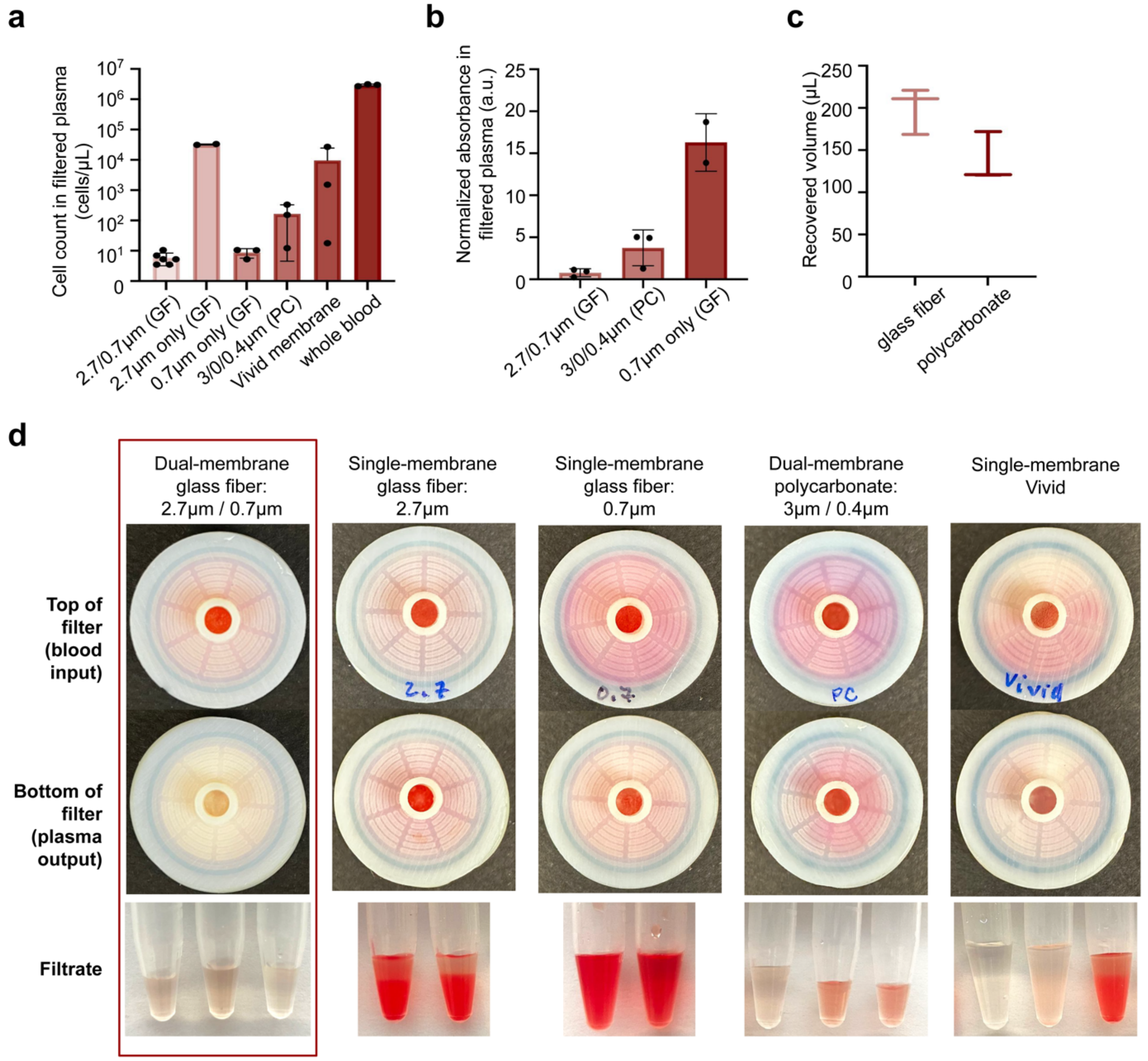

Supplemental Figure 1. Plasma separation filter characterization: membrane selection. (a) Plasma separation performance for filter with various combinations of single or dual membranes. Dual membrane filtration with 2.7 μm and 0.7 μm pore size glass fiber membranes demonstrated the highest filtration efficiency indicated by low cell counts remaining in filtered plasma, compared to single glass fiber membranes, dual polycarbonate membranes (3.0 μm and 0.4 μm), and the Vivid plasma separation membrane. **(b)** Evaluation of hemolysis in plasma filtered by membranes with various combinations of single or dual membranes. Dual membrane filtration with 2.7 μm and 0.7 μm pore size glass fiber membranes demonstrated the lowest degree of hemolysis, indicated by low absorbance at 414 nm, compared to dual polycarbonate membranes and a single 0.7 μm glass fiber membrane. **(c)** Recovered volume of plasma filtered by dual glass fiber (2.7 μm and 0.7 μm) and polycarbonate membranes (3.0 μm and 0.4 μm). The dual glass fiber design yielded a higher average volume of 201.8 μL ± 18.75 μL, while filtration with dual polycarbonate membranes resulted in 137.9 ± 24.13 μL of filtrate. **(d)** Images of filter input, output, and filtrate

for various combinations of dual and single membranes. Dual glass fiber membranes demonstrated high red blood cell filtration and low hemolysis, indicated by the red color at the filter input showing successful retention of red blood cells in the membranes with a clear yellow color at the output and filtrate. Single 2.7 μm and Vivid membranes showed limited filtration, shown by the appearance of red blood cells in the filtrate. Dual polycarbonate membranes and single 0.7 μm membranes resulted in higher hemolysis, indicated by the red tint in the filtrate and at the filter outputs.

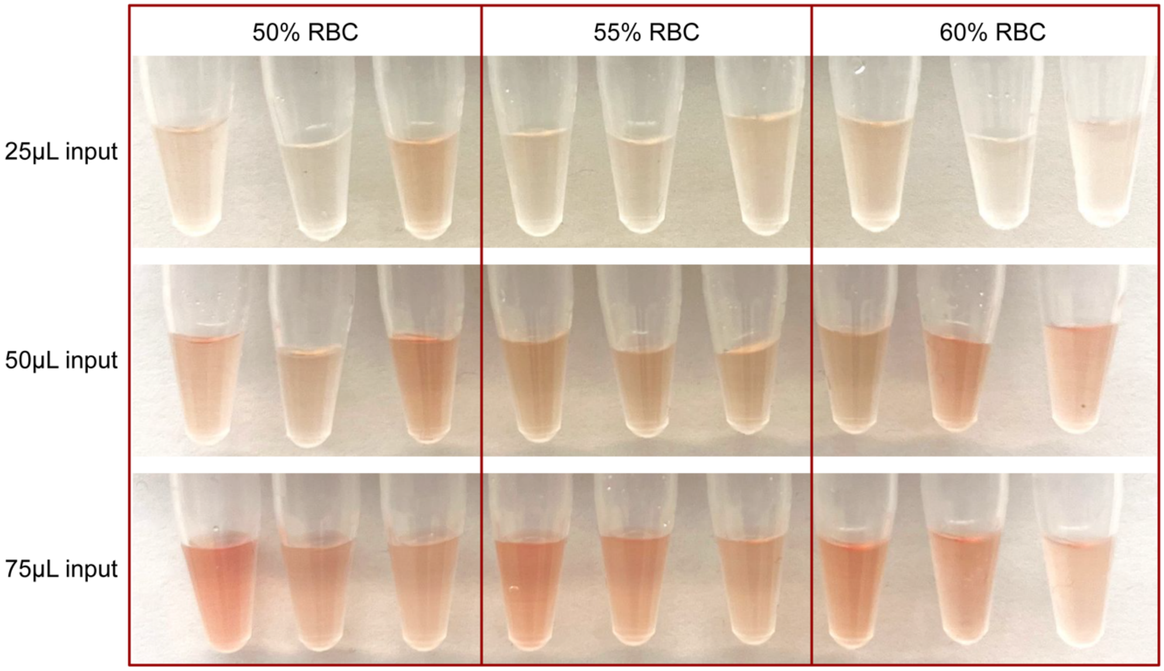

Supplemental Figure 2. Filtrate images at varying hematocrit and sample volume levels. Images of resulting filtrate from filtration of blood at various hematocrit levels and input volumes in the filter. There is an observable trend in increasing hemolysis at higher input volumes, as indicated by visually darker filtrate with a red tint. Filtrate is visually consistent across hematocrit levels for the same volumes, quantified by no significant difference in hemolysis between hematocrit levels (Fig. 2i).

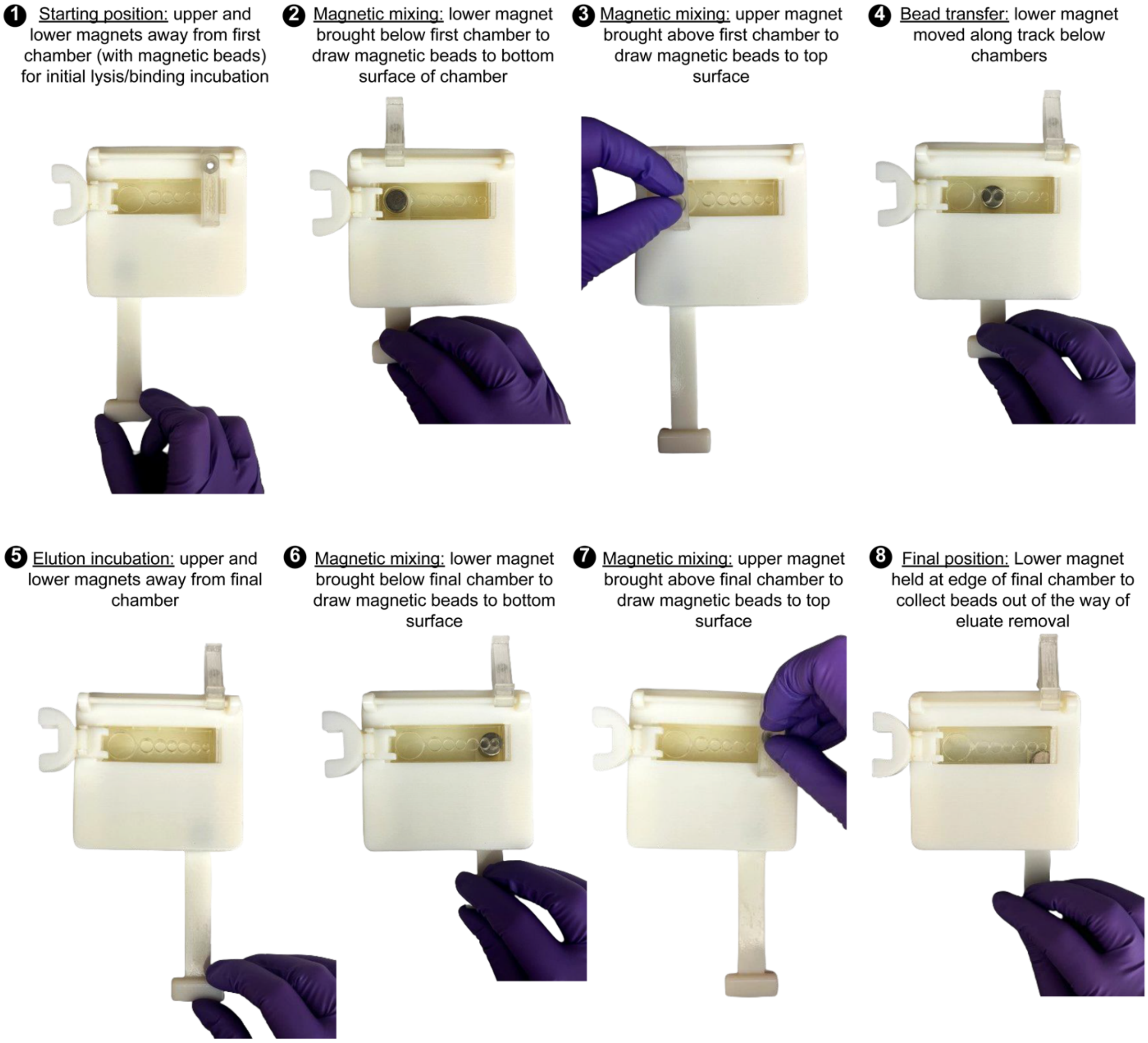

Supplemental Figure 3. Power-free, manually operated guided magnetic track of PRECISE device. Still images of all possible manual magnet movements by the user. The upper magnet moves vertically with respect to the extraction module to resuspend magnetic beads in the chamber, while the upper magnetic track moves horizontally to move the mixing magnet between the lysis/binding and elution chambers. The lower magnet moves vertically to draw and release magnetic beads and horizontally to transfer magnetic beads between chambers.

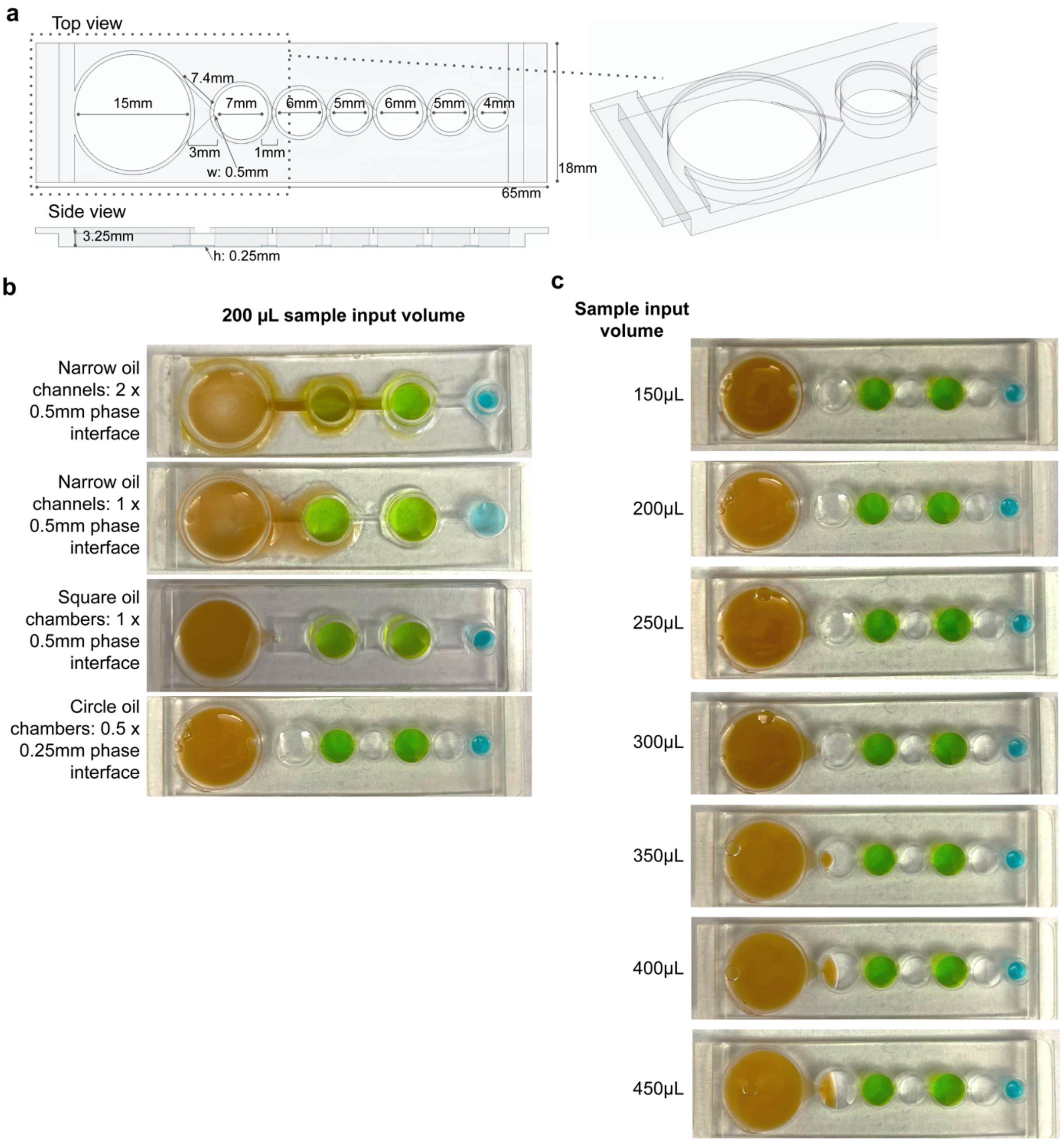

Supplemental Figure 4. Fluidic characterization in nucleic-acid extraction module containing different geometries of microchannels and chambers. (a) Schematic of oil/reagent interface design. Design includes triangular narrowing between chambers to help guide beads into the next chamber. Interface between phases has 0.5 mm width and 0.25mm height; this design was found to be most tolerant of containing larger volumes without breaking the barrier between lysis chamber reagent mix and the mineral oil. Chamber dimensions (shown in figure) varied depending on necessary volume of reagents for each step in extraction protocol. **(b)** Images of extraction module with various oil channel geometries at 200 µL of sample input volume: lysis chamber contains brown-colored liquid (due to magnetic beads in suspension), wash chambers are indicated with green dye, and elution chamber indicated with blue dye;

mineral oil is transparent. Earlier design iterations revealed that increasing volumes of liquid in the lysis/binding chamber resulted in leakage of reagents into the oil phase, owing to the significantly lower surface tension of lysis buffers containing detergents compared to water (1). As discussed by previous studies utilizing immiscible phase separation (2), the separation of the aqueous and oil phases during device loading relies upon the dominance of surface tension over gravity. This relationship can be quantified using Bond number ($Bo = \rho g L^2/\gamma$), and $Bo \ll 1$ indicates that surface tension forces dominate the effects of gravity; the L^2 term (characteristic length, or phase interface area at the barrier between oil and reagent in this case) dominates this relationship, thus we aimed to minimize L in order to minimize Bo. Experimentally, observations demonstrated phase interface influenced how well the barrier was maintained without leaking for the lysis chamber. We first tested decreasing sizes of phase interfaces, and found that smaller interfaces better maintained the barrier (module design with larger interface 2 x 0.5mm (top image) leaked more severely than 1 x 0.5mm design (second to top image), when adding 200µL sample volume). Additionally, oil volume held in the oil chamber appeared to be important in maintaining the interface. Square oil chambers holding 100µL oil, compared to 20-30µL oil in previous, with 1 x 0.5mm interface (second to bottom image) did not leak at 200µL sample volume, yet began to show signs of encroachment of the oil barrier at this sample volume, likely indicating that 200 µL approaches the lysis chamber's volume capacity for that geometry with a larger phase interface. Thus, combining the concepts of minimizing phase interface and increasing oil volume, we created a circular oil chamber design to hold 100µL oil with a 0.5 x 0.25mm phase interface (bottom image) that could accommodate 200 µL without leaking or signs of encroachment into the oil. **(c)** Images of extraction module design chosen in (b) tested for tolerance of various input sample volumes. The lysis/binding chamber can accommodate a sample volume of up to 300 µL without leaking into the first oil chamber, which is well within the expected yield of the filter (200µL average yield, see Fig. 2f). Sample volumes up to 450 µL can be added without leaking into subsequent reagent chambers.

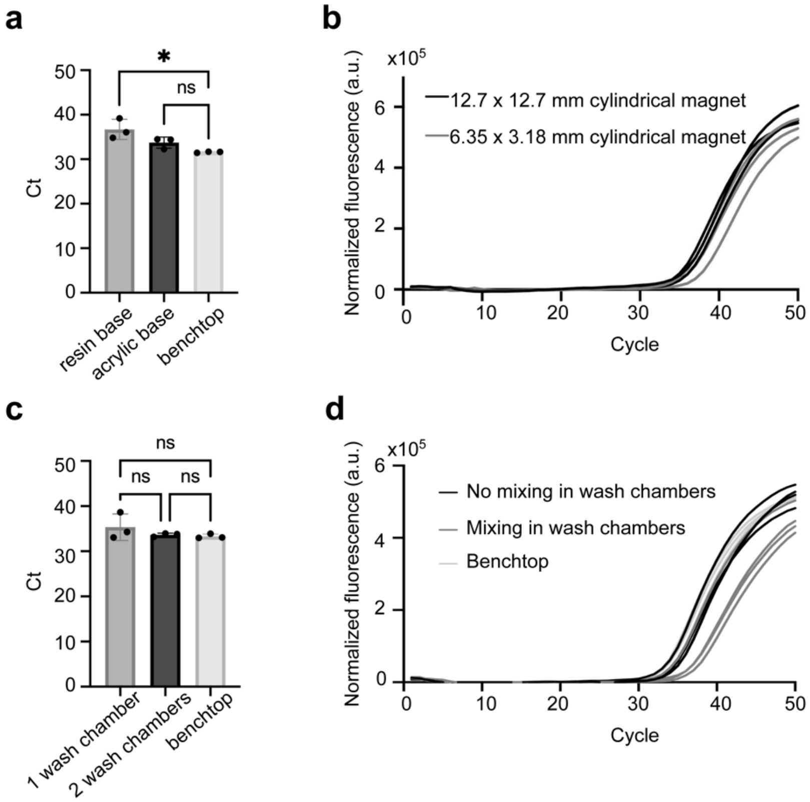

Supplemental Figure 5. Optimization of on-cartridge nucleic acid extraction. (a) Evaluation of the effect of various bottom surfaces on cartridge RNA extraction performance by PCR C_t. There is higher variability in RNA extraction performance on cartridges with 3D-printed resin bases, likely due to variable surface roughness during DLP 3D-printing and post-processing. Cartridges with acrylic (PMMA) bases show similar performance to benchtop RNA extraction (p=0.2677) while there is a significant difference in C_t between benchtop extraction and cartridge extraction with a resin base (p=0.0139). **(b)** PCR amplification curves of cartridge-extracted RNA using various magnet sizes. The 12.7 x 12.7 mm cylindrical magnet demonstrated slightly earlier amplification than the smaller 6.35 x 3.18 mm cylindrical magnet, potentially due to decreased magnetic bead loss due to stronger magnetic field. **(c)** Evaluation of the effect of number of wash chambers on RNA extraction performance. There was no significant difference in cartridges with one wash chamber vs. two wash chambers (p = 0.4999), but there was more variability in C_t in cartridges with only one wash chamber. **(d)** PCR amplification curves of cartridge-extracted RNA with and without on-cartridge mixing in the wash chambers. Mixing in the wash chambers resulted in delayed amplification compared to cartridge extraction runs where there was no mixing, which was similar in performance to benchtop RNA extraction.

SUPPLEMENTAL REFERENCES

1. Snyder SA, Boban M, Li C, VanEpps JS, Mehta G, Tuteja A. Lysis and direct detection of coliforms on printed paper-based microfluidic devices. Lab Chip. 2020 Nov 24;20(23):4413–9.
2. Berry SM, Alarid ET, Beebe DJ. One-step purification of nucleic acid for gene expression analysis via Immiscible Filtration Assisted by Surface Tension (IFAST). Lab Chip. 2011 May 21;11(10):1747–53.
